# Linalyl acetate alleviates inflammatory pain via OLFR482 activation in the mouse anterior cingulate cortex 

**DOI:** 10.3389/fphar.2026.1867848

**Published:** 2026-07-03

**Authors:** Ban Feng, Wei Guo, Shu Zhao, Han Wang, Xinshang Wang, Kun Zhang, Dake Song, Peng Gao

**Affiliations:** 1 Shanghai Key Laboratory of Anaesthesiology and Brain Functional Modulation, Translational Research Institute of Brain and Brain-Like Intelligence, Department of Anaesthesiology and Perioperative Medicine, Clinical Research Center for Anaesthesiology and Perioperative Medicine, Shanghai Fourth People’s Hospital, School of Medicine, Tongji University, Shanghai, China; 2 Department of Pediatrics, Tangdu Hospital, Fourth Military Medical University, Xi’an, China; 3 Department of Pharmacology, School of Pharmacy, Fourth Military Medical University, Xi’an, China; 4 State Key Laboratory of Military Stomatology, Department of Pharmacy, National Clinical Research Center for Oral Diseases, Shaanxi International Joint Research Center for Oral Diseases, School of Stomatology, Fourth Military Medical University, Xi’an, China

**Keywords:** anterior cingulate cortex, chronic inflammatory pain, linalyl acetate, neuronal excitability, olfactory receptors

## Abstract

**Introduction:**

Chronic pain severely impairs quality of life and causes heavy socioeconomic burdens worldwide. Linalyl acetate (LA), a major volatile terpenoid ester from essential oils, possesses potential analgesic effects.

**Methods:**

A CFA-induced chronic inflammatory pain mouse model was established. Behavioral tests, immunofluorescence, whole-cell patch-clamp recording, RNA-seq, molecular docking, molecular dynamics simulation and AAV-mediated gene knockdown were applied to explore the analgesic mechanism of LA.

**Results:**

Systemic LA administration significantly alleviated inflammatory pain and suppressed neuronal hyperexcitability in the anterior cingulate cortex (ACC). Transcriptome analysis revealed marked upregulation of Olfr482 in the ACC of CFA-treated mice, and molecular docking confirmed the interaction between LA and OLFR482. Olfr482 knockdown largely abolished the analgesic effect of LA.

**Discussion:**

LA relieves chronic inflammatory pain via an olfactory-independent mechanism by activating OLFR482 and reducing neuronal hyperexcitability in the ACC. LA is a promising candidate for developing novel analgesic drugs.

## Introduction

The experience of pain involves intricate physiological and emotional dimensions, exerting a profound influence on human wellness and daily function. With advances in modern medicine, pain management has become an indispensable component of clinical care. Nevertheless, while conventional analgesics such as opioids exhibit powerful pain-relieving activity, their clinical translation is greatly hampered by untoward effects, notably addiction, tolerance, and additional complications linked to long-term usage (Coles et al., 2022; Carroll and Brandow, 2022). Therefore, the identification of novel, safe, and effective analgesic compounds and their underlying mechanisms remains a high priority. The anterior cingulate cortex (ACC) acts as an essential node for integrating sensory input with affective responses related to pain. Its dysfunction is tightly linked to the initiation and maintenance of both acute and chronic pain states (Bliss et al., 2016). In particular, the ACC plays an essential role in encoding nociceptive signals and modulating pain-related behaviors (Li et al., 2010), positioning it as a promising target for developing mechanism-based analgesic therapies.

Linalyl acetate (LA) is a representative volatile terpenoid ester, which can account for up to 35% of the volatile components in essential oils derived from Salvia sclarea and Lavandula angustifolia (Seol et al., 2013). Widely used as a flavor and fragrance ingredient in personal care products, detergents, and cosmetics (Letizia et al., 2003), LA is also safely consumed orally, for instance, approximately 0.2 mg is present in a single cup of Earl Grey tea, contributing to its characteristic bergamot aroma (Orth et al., 2014). Emerging evidence indicates that LA exhibits analgesic properties with high safety profiles and may act through non-olfactory pathways (Koulivand et al., 2013). For example, intraperitoneal LA administration suppresses spinal glial inflammation and elevates mechanical pain thresholds in rat model of sciatic nerve injury (Lu Y. Y. et al., 2022). However, the precise central mechanisms underlying its olfactory-independent analgesic action remain largely unknown.

Olfactory receptors (OLFRs) are transmembrane G protein-coupled receptors (GPCRs) canonically distributed in olfactory sensory neurons of the nasal epithelium, where they detect odorants and translate chemical cues into electrical neuronal responses (Francia and Lodovichi, 2021). Since the first report of ectopic OLFR expression in mammalian germ cells in 1992, numerous family members have been identified in non-olfactory tissues including testis, skin, adipose tissue, and intestine (He and Wang, 2022; Ra et al., 2022; Jundi et al., 2023). For instance, OLFR78 is expressed in vasopressin/oxytocin neurons of the hypothalamic paraventricular and supraoptic nuclei, as well as in perivascular microglia (Nakashima et al., 2022). Ectopic OLFRs participate in an array of physiological events, encompassing sperm navigation, tissue restoration, control of sugar and fat metabolism, and gastrointestinal secretion (Galibert and Azzouzi, 2023; Geng et al., 2023; Zhang et al., 2021; Meijerink, 2021), and are also implicated in cancer cell proliferation, apoptosis, invasion, and metastasis (Yang et al., 2024). These diverse functions highlight ectopic OLFRs as attractive and tractable therapeutic targets.

In the present study, we investigated whether LA produces analgesia through an olfactory-independent mechanism. We found that systemic LA administration significantly alleviated chronic inflammatory pain in mice. Mechanistically, LA attenuated neuronal hyperexcitability in the ACC. High-throughput RNA sequencing revealed marked upregulation of *Olfr482* in the ACC of CFA-treated mice, and molecular docking predicted a putative interaction between LA and OLFR482. Importantly, AAV-mediated shRNA knockdown of *Olfr482* in the ACC abolished LA-induced analgesia. Together, our findings demonstrate that LA exerts potent analgesic effects via an ACC-restricted, OLFR482-dependent pathway independent of the canonical olfactory system.

## Results

### Systemic administration of LA alleviates CFA-induced chronic inflammatory pain

**FIGURE 1:**
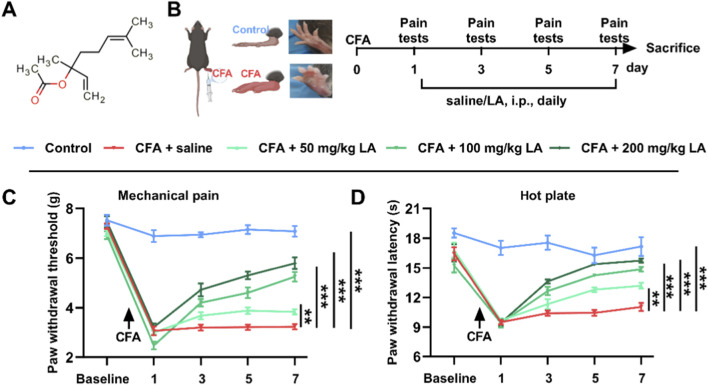
Systemic administration of LA produce analgesic effects. **(A)** The chemical structure of LA. **(B)** The schedule of the experimental design. **(C)** Effect of different concentrations of LA on mechanical pain threshold according von-frey test. Two-way repeated-measures ANOVA, Bonferroni’s *post hoc* test: CFA + saline vs. Control, *P* < 0.001, CFA+50 mg/kg LA vs. CFA + saline, *P* = 0.005, CFA+100 mg/kg LA vs. CFA + saline, *P* < 0.001, CFA+200 mg/kg LA vs. CFA + saline, *P* < 0.001. **(D)** Effect of different concentrations of LA on thermal pain threshold according to hot plate test. Two-way repeated-measures ANOVA, Bonferroni’s *post hoc* test: CFA + saline vs. Control, *P* < 0.001, CFA+50 mg/kg LA vs. CFA + saline, *P* = 0.0033, CFA+100 mg/kg LA vs. CFA + saline, *P* < 0.001, CFA+200 mg/kg LA vs. CFA + saline, *P* < 0.001. Data are shown as mean ± s. e.m. n = 8 mice per group.

Building on prior evidence that lavender essential oil exerts analgesic effects in humans and mice (Yang et al., 2024; Khamis et al., 2023; Sanna et al., 2019), we examined whether systemic LA, a major constituent of lavender oil, produces antinociceptive effects in mice. A chronic inflammatory pain model was generated by intraplantar CFA injection into the mouse hind paw, which reliably elicited long-lasting mechanical and thermal hyperalgesia (Qin et al., 2024). The chemical structure of LA is illustrated in Figure 1A. CFA-treated mice were administered LA intraperitoneally at doses of 50, 100, or 200 mg/kg once daily for seven consecutive days (Figure 1B). The three doses were selected to establish a dose-response relationship based on the results reported in the literature (Hashimoto et al., 2024a; Hashimoto et al., 2024b; Lu Y.-Y. et al., 2022). As shown in Figure 1C, mechanical pain threshold was markedly reduced in CFA-treated mice, and LA administration dose-dependently restored mechanical sensitivity. Similarly, LA dose-dependently reversed CFA-induced thermal hyperalgesia (Figure 1D). These results demonstrate that systemic LA effectively attenuates CFA-evoked chronic inflammatory pain in mice.

### LA suppresses CFA-induced c-Fos activation in the ACC

**FIGURE 2:**
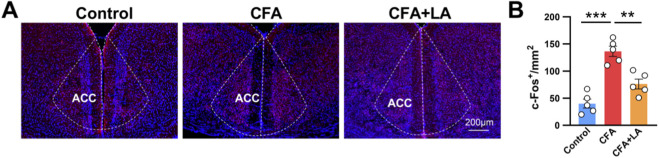
LA suppresses CFA-induced c-Fos activation in the ACC. **(A)** Representative imagines of c-Fos staining in the ACC (Red, c-Fos; Blue, cell nucleus). **(B)** Counting results of c-Fos positive (c-Fos^+^) neurons of different groups. One-way ANOVA, Tukey’s *post hoc* test: CFA vs. Control, *P* < 0.001; CFA vs. CFA + LA, *P* = 0.0013. n = 5.

To identify brain regions underlying LA-mediated analgesia, we performed c-Fos immunofluorescence as a marker of neuronal activation (Song et al., 2025). CFA injection robustly increased c-Fos expression in multiple brain regions, with the most prominent induction observed in the ACC (Figure 2A), a key region for pain processing and modulation. Of note, systemic delivery of 100 mg/kg LA significantly attenuated the number of c-Fos–immunoreactive neurons within the ACC in CFA-challenged mice (Figure 2A). Quantification confirmed that LA reversed CFA-induced c-Fos upregulation (Figure 2B). These data indicate that LA alleviates inflammatory pain at least partially by inhibiting neuronal hyperactivity in the ACC.

### LA inhibits excitability of ACC layer II/III pyramidal neurons

**FIGURE 3:**
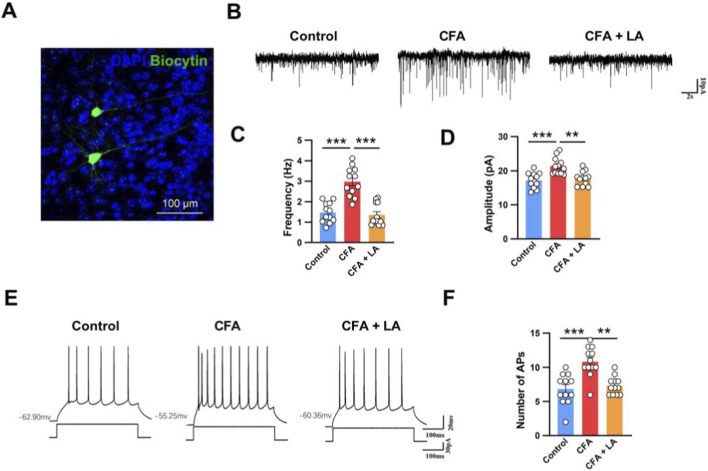
LA inhibits the excitability of pyramidal neurons in layers II/III of the ACC in mice. **(A)** Schematic diagram of pyramidal neurons in the ACC (green, indicating recorded neurons). **(B–D)** Intraperitoneal administration of LA can significantly reverse the increased frequency **(C)** and amplitude **(D)** of sEPSCs in mice with inflammatory pain model. n = 12 neurons from six mice per group. Frequency: One-way ANOVA, Tukey’s *post hoc* test: CFA vs. Control, *P* < 0.001, CFA + LA vs. CFA, *P* < 0.001; amplitude: One-way ANOVA, Tukey’s *post hoc* test: CFA vs. Control, *P* = 0.0002, CFA + LA vs. CFA, *P* = 0.0013. **(E,F)** Inflammatory pain model mice injected with LA can significantly reduce the number of APs of pyramidal neurons in layers II/III of the ACC. One-way ANOVA, Tukey’s *post hoc* test: CFA vs. Control, *P* < 0.001; CFA + LA vs. CFA, *P* = 0.003. n = 12 per group.

Accumulating evidence indicates that pyramidal neuron firing in the ACC encodes noxious sensory inputs and critically contributes to pain-related affective and cognitive dimensions (Kuai et al., 2026). As increased neuronal excitability, reflected by elevated c-Fos expression, is a hallmark of chronic pain pathogenesis, we conducted whole-cell patch-clamp recordings to specifically target layer II/III pyramidal neurons (Figure 3A)—these represent the primary recipients and integrators of nociceptive signals within the ACC. These recordings were performed on ACC slices obtained from mice 30 min after systemic LA injection (100 mg/kg), thus reflecting the integrated effects of *in vivo* LA administration. Compared with controls, CFA-treated mice exhibited significant increases in both the frequency and amplitude of spontaneous excitatory postsynaptic current (sEPSC) (Figures 3B–D), indicating enhanced presynaptic glutamate release and postsynaptic responsiveness. LA administration normalized both sEPSC frequency and amplitude. Current-clamp recordings further showed that CFA-induced increases in evoked action potential firing were markedly suppressed by LA (Figures 3E,F). These results demonstrate that LA directly reduces hyperexcitability of ACC pyramidal neurons in inflammatory pain.

### *Olfr482* is upregulated in the ACC of CFA-treated mice and is predicted to interact with LA

**FIGURE 4:**
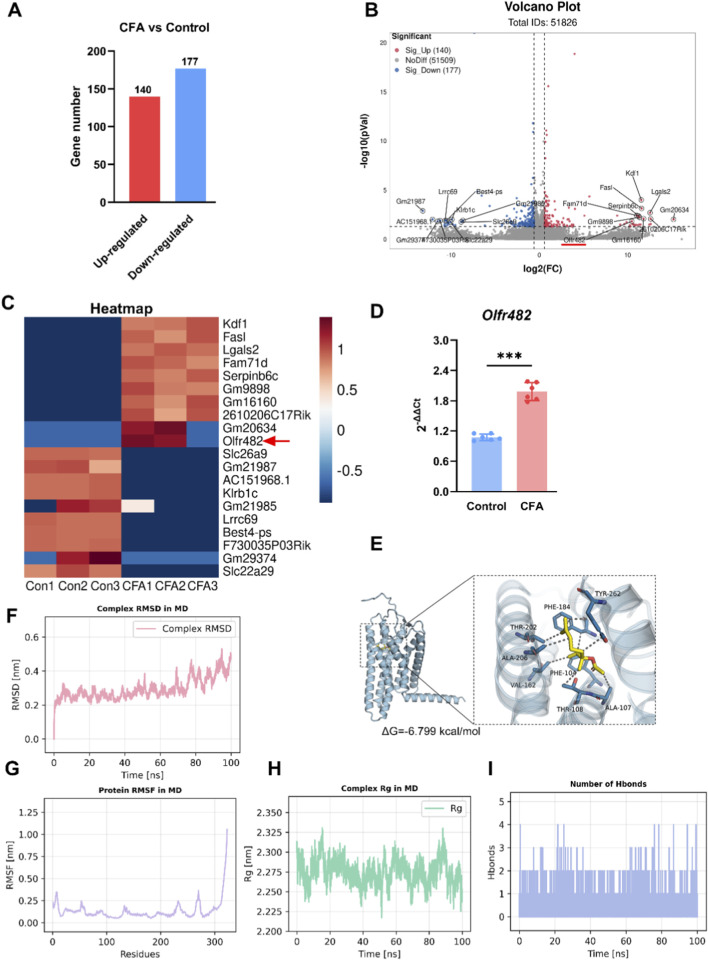
Transcriptome sequencing reveals that the expression of *Olfr482* in the ACC of mice with an inflammatory pain model is significantly increased. **(A)** Statistics of the number of differentially expressed genes obtained from transcriptome sequencing. **(B)** Volcano plot of the transcriptome sequencing results, with the top 10 upregulated and downregulated genes labeled. **(C)** Heatmap analysis of the top 10 differentially expressed genes, including both upregulated and downregulated genes. **(D)** qRT-PCR verifies that the mRNA level of *Olfr482* in the ACC brain region increases significantly. Unpaired t-test: t_10_ = 11.84, *P* < 0.001. n = 6 mice per group. **(E)** Molecular docking predicting the interaction between LA and OLFR482. Left: overall complex; right: close-up view. LA is shown as yellow sticks, OLFR482 as cyan cartoon. **(F–I)** Molecular dynamics simulation of the LA–OLFR482 complex over 100 ns. **(F)** Complex RMSD (protein + ligand), **(G)** Protein RMSF (root-mean-square fluctuation) per residue, **(H)** Radius of gyration (Rg) of the complex, **(I)** Number of hydrogen bonds between LA and OLFR482. All data are derived from a 100-ns MD simulation using AMBER 24.

To explore molecular mediators in the ACC, we performed RNA-seq on ACC tissues from control and CFA-injected mice. Transcriptomic analysis identified 140 upregulated and 177 downregulated genes (Figure 4A). Volcano and heatmap plots revealed marked upregulation of *Olfr482*, an olfactory receptor family member (Figures 4B,C). Real-time quantitative PCR (RT-qPCR) further validated that *Olfr482* expression was significantly upregulated in the ACC following CFA treatment (Figure 4D). To explore whether LA can directly interact with OLFR482, we performed molecular docking using AutoDock Vina. The predicted binding affinity between LA and OLFR482 was −6.8 kcal/mol (Figure 4E). To assess the stability of this interaction, we conducted a 100 ns molecular dynamics simulation using AMBER 24 (Figures 4F–I). As shown in Figure 4F, the complex RMSD rapidly increased to 0.22–0.28 nm in the first few nanoseconds and remained relatively stable at 0.25–0.32 nm during 0–70 ns, but gradually increased with notable fluctuations after ∼75 ns, peaking near 0.50 nm, suggesting a conformational rearrangement or transition to a metastable state in the later phase. The protein RMSF was low (<0.2 nm) for most residues, with only terminal regions showing higher flexibility (Figure 4G). The radius of gyration (Rg) remained stable throughout the simulation (Figure 4H), indicating that the complex did not undergo unfolding or global conformational collapse. Hydrogen bonds between LA and OLFR482 formed intermittently (0–4 bonds), supporting dynamic but persistent polar interactions (Figure 4I). Collectively, these results identify OLFR482 as a candidate target for LA in the ACC.

### Analgesic effects of LA require OLFR482 in the ACC

**FIGURE 5:**
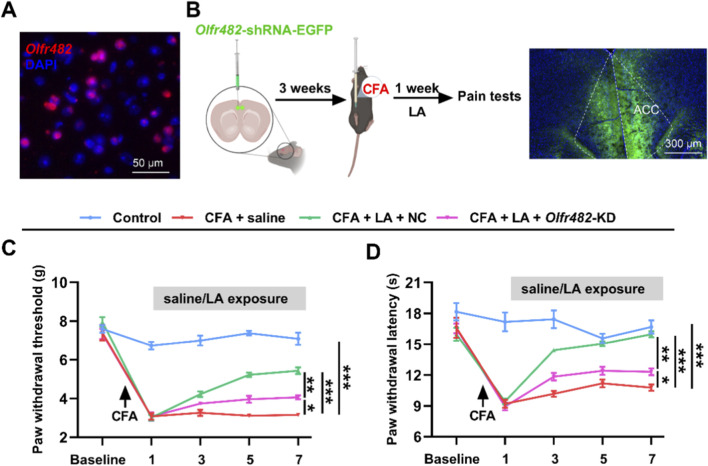
Knocking down *Olfr482* in the ACC nearly abolishes the analgesic effect of LA. **(A)** Representative image showing the *Olfr482* mRNA expression in the ACC. **(B)** Schematic diagram of viral injection in the ACC brain region and the experimental protocol. **(C)** Mechanical pain threshold measured by electronic von Frey test. Two-way repeated-measures ANOVA, Bonferroni’s *post hoc* test: CFA + saline vs. Control, *P* < 0.001; CFA + LA + NC vs. CFA + saline, *P* < 0.001; CFA + LA + *Olfr482-KD* vs. CFA + LA + NC, *P* = 0.009; CFA + LA + *Olfr482-KD* vs. CFA + saline, *P* = 0.012. **(D)** Thermal pain threshold measured by hot plate test (paw withdrawal latency). Two-way repeated-measures ANOVA, Bonferroni’s *post hoc* test: CFA + saline vs. Control, *P* < 0.001; CFA + LA + NC vs. CFA + saline, *P* < 0.001; CFA + LA+ *Olfr482-KD* vs. CFA + LA + NC, *P* = 0.0053; CFA + LA+ *Olfr482-KD* vs. CFA + saline, *P* = 0.034. n = 8 mice per group.

To determine whether OLFR482 mediates LA-induced analgesia, we first confirmed *Olfr482* expression in the ACC by fluorescence *in situ* hybridization (FISH) (Figure 5A). We subsequently employed AAV-mediated shRNA to achieve bilateral *Olfr482* knockdown in the ACC. Three weeks later, mice were subjected to CFA injection, followed by LA administration and subsequent behavioral nociceptive tests (Figure 5B). Strikingly, *Olfr482* knockdown nearly abolished the analgesic effects of LA on both mechanical and thermal hyperalgesia (Figures 5C,D). These findings demonstrate that OLFR482 in the ACC is essential for LA-mediated antinociception.

### LA inhibits ACC neuronal excitability via an OLFR482-dependent mechanism

**FIGURE 6:**
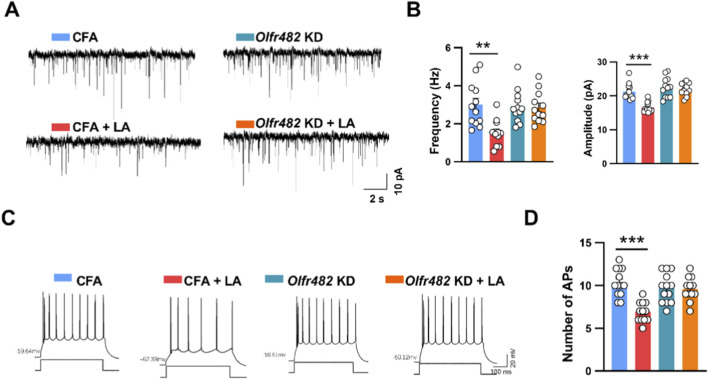
*Olfr482* knockdown abolishes the inhibitory effect of LA on pyramidal neurons in CFA mice. **(A)** Representative traces illustrating the effect of LA on sEPSCs in ACC pyramidal neurons following *Olfr482* KD. **(B)** Quantification of sEPSC frequency (left) and amplitude (right). Frequency: Two-way ANOVA, Bonferroni’s *post hoc* test: CFA + LA vs. CFA, *P* = 0.0028. Amplitude: Two-way ANOVA, Bonferroni’s *post hoc* test: CFA + LA vs. CFA, *P* < 0.001. **(C,D)** Representative traces **(C)** and quantitative summary **(D)** of APs in ACC pyramidal neurons before and after LA application in *Olfr482* KD mice. Two-way ANOVA, Bonferroni’s *post hoc* test: CFA + LA vs. CFA, *P* < 0.001. n = 12.

Our previous results demonstrated that LA effectively reversed the elevated excitability of ACC pyramidal neurons in CFA-treated mice. We next explore whether OLFR482 mediates LA’s inhibitory effects on ACC excitability. Electrophysiological recordings revealed that LA no longer reduced the frequency or amplitude of sEPSCs in ACC pyramidal neurons after *Olfr482* knockdown (Figures 6A,B). Consistently, following *Olfr482* knockdown, LA no longer exerted its suppressive effect on evoked action potential firing (Figures 6C,D). These data establish that OLFR482 is required for LA-mediated attenuation of neuronal hyperexcitability in the ACC during inflammatory pain.

## Discussion

A limited number of existing clinical randomized controlled trials have demonstrated that nasal inhalation of LA effectively alleviates various types of pain (You et al., 2024; Yu and Seol, 2017). Computational modeling further indicates that LA does not act as a substrate or inhibitor of cytochrome P450 enzymes, such as CYP1A2, CYP2D6, CYP3A4, thereby minimizing concerns regarding pharmacokinetic interactions with neuropsychiatric medications (Avram et al., 2021). Previous studies have shown that LA can also exert peripheral analgesic effects by inhibiting TRPA1 and TRPV1 channels (Hashimoto et al., 2024a; Hashimoto et al., 2024b). Here, we extend these findings by demonstrating that systemic administration of LA alleviates CFA-induced inflammatory pain through a predicted interaction with OLFR482 in the ACC. This work expands the current understanding of LA’s central nervous system mechanisms and identifies a novel olfactory receptor-mediated pathway underlying its analgesic action.

Beyond LA, numerous essential oil constituents have been documented to exhibit analgesic properties, highlighting the potential of plant-derived compounds as alternative analgesics. For instance, linalool, another major component of many essential oils, significantly elevates pain thresholds and attenuates formalin-induced nociceptive responses by activating hypothalamic orexin neurons (Higa et al., 2021; Iida et al., 2024). Additionally, Lippia grata leaf essential oil reduces orofacial pain in mice by modulating neuronal activity in the nucleus raphe magnus and periaqueductal gray (PAG), key nodes in the descending pain modulation pathway (Siqueira-Lima et al., 2017; Siqueira-Lima et al., 2014). A favorable safety profile is critical for the clinical translation of analgesic agents, and toxicological assessments have consistently confirmed LA’s safety. Repeated-dose toxicity studies in rodents revealed no adverse effects at daily oral doses of 36 mg/kg, while reproductive toxicity evaluations showed no teratogenic or embryotoxic effects at doses up to 200 mg/kg/day (Api et al., 2015). In a human clinical trial, daily oral administration of lavender essential oil (containing approximately 30% LA) for 2 weeks exerted no significant effects on plasma lipid profiles or hepatic enzyme levels in healthy athletes, thus supporting its favorable safety profile for short-term oral application (Maral et al., 2022). Collectively, these results further reinforce LA’s safety profile and its potential as a clinically viable analgesic candidate.

Our study identifies a novel role for LA in alleviating inflammatory pain in a murine model. However, several limitations merit consideration and future investigation. We demonstrated robust functional effects of LA in the ACC, but we did not explore the contributions of other key pain-modulating brain regions, such as the PAG and somatosensory cortex (Subramanian and Holstege, 2014; Soldatelli et al., 2023). These regions form interconnected neural circuits with the ACC to regulate pain perception, and future studies should investigate whether LA exerts coordinated effects across these networks. Additionally, all the experiments in this study were performed exclusively in male mice. However, accumulating evidence has demonstrated robust sex differences in pain processing, inflammatory responses, and analgesic mechanisms in rodents and humans (Silveira Prudente et al., 2024; Sorge et al., 2015; Lee et al., 2023; Mogil, 2012; Sorge et al., 2015). Therefore, our findings obtained in male mice may not fully generalize to females, and the efficacy and mechanism of LA in female mice remain to be determined. Future studies using both male and female animals are strongly warranted to validate the analgesic effects of LA and characterize potential sex-dependent differences in OLFR482 signaling in the ACC.

Although our loss-of-function experiments establish that OLFR482 is necessary for LA-induced analgesia and suppression of ACC pyramidal neuron excitability, the intracellular signaling cascades downstream of OLFR482 activation were not directly investigated in this study. Based on well-established olfactory receptor signaling and GPCR biology, we propose two plausible downstream pathways that may mediate OLFR482-dependent inhibition of ACC pyramidal neurons. Many olfactory receptors signal through the Gα_olf_ subunit, which activates adenylate cyclase to increase cAMP levels. Elevated cAMP can activate cyclic nucleotide-gated (CNG) channels and trigger Ca^2+^ influx (Jones and Reed, 1989; Pluznick et al., 2009; Belluscio et al., 1998). In ACC pyramidal neurons, this Ca^2+^ transient may activate Ca^2+^-dependent K^+^ channels (SK/CaBK channels) to hyperpolarize neurons and reduce excitability, consistent with our electrophysiological findings that LA suppresses action potential firing and sEPSCs. Additionally, upon GPCR activation, the Gβγ dimer can directly bind and open G-protein-inwardly rectifying K^+^ (GIRK) channels, leading to K^+^ efflux and membrane hyperpolarization. GIRK channels are highly expressed in cortical pyramidal neurons and strongly inhibit neuronal excitability (Lüscher and Slesinger, 2010; Ted et al., 2006). We hypothesize that OLFR482 activation by LA releases Gβγ subunits to enhance GIRK currents, thereby reducing hyperexcitability in the ACC during inflammatory pain. These two pathways are strongly supported by previous studies of ectopic olfactory receptors and cortical GPCR signaling. Future studies using cAMP ELISA, Ca^2+^ imaging, GIRK channel pharmacology, and Gα_olf_/Gβγ perturbation will be needed to verify these signaling cascades and fully define the molecular mechanism of LA-OLFR482 analgesia.

Although our molecular docking analysis suggested direct interaction between LA and OLFR482, subsequent studies are warranted to verify this binding using biochemical assays and to characterize the downstream signaling cascades triggered by LA-OLFR482 engagement in ACC neurons.

**FIGURE 7:**
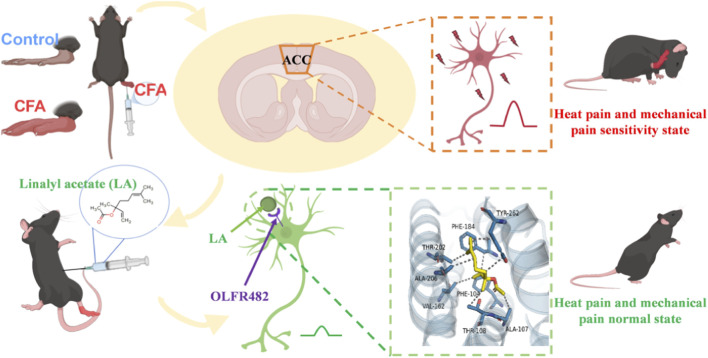
Intraperitoneal administration of LA alleviates chronic inflammatory pain through an olfactory system-independent pathway. LA binds to OLFR482 in the ACC to suppress neuronal excitability, thereby identifying LA as a promising candidate for the development of novel therapeutic strategies against chronic inflammatory pain.

In summary, systemic LA treatment relieves CFA-induced chronic inflammatory pain through an olfactory-independent mechanism by targeting ACC OLFR482 and reducing neuronal hyperexcitability. Our study identifies OLFR482 as a potential new molecular target of LA and provides mechanistic insights into the central analgesic effects of plant-derived small molecules. With its good safety and analgesic efficacy, LA is a promising lead compound for developing novel therapies against chronic inflammatory pain. The schematic mechanism is illustrated in Figure 7.

## Materials and methods

### Materials

LA was purchased from MedChemExpress (catlog: HY-N6948, Shanghai, China), and the purity was no less than 98%, as determined by high-performance liquid chromatography. CFA was purchased from Sigma–Aldrich (catlog: F5881St. Louis, MO, USA).

### Animals

Male C57BL/6 J mice (6–8 weeks, 20.0 ± 2.0 g) were obtained from the Laboratory Animal Center of Tongji University. After acclimatization, mice were randomly grouped with investigators blinded to treatments. Chronic inflammatory pain was induced by intraplantar injection of 10 μL 50% CFA into the right hind paw; control mice received saline only. All procedures were approved by the Institutional Animal Care and Use Committee of Tongji University (IACUC-20221227). Animals were managed to minimize usage and suffering.

### Mechanical allodynia test

Animals were placed inside individual clear enclosures atop a wire mesh platform and allowed a 30-min adaptation period. From underneath the mesh, hold the electronic von Frey probe (Yuyan Instruments, Shanghai, China) vertically and align its tip perpendicularly to the center of the mouse’s hind paw plantar surface. Apply a steadily increasing pressure at a consistent rate. Closely observe the mouse for a clear nocifensive behavior, such as paw withdrawal, licking, or flinching. Immediately stop applying pressure upon observing such a response. The instrument will automatically record the peak pressure value. Simultaneously, press the foot switch or manually record the value. To ensure data reliability, repeat the test 3 to 5 times per hind paw for each mouse. Allow an inter-stimulus interval of at least 30 s to 2 min to avoid pain fatigue or sensitization.

### Thermal hyperalgesia test

Mice were acclimated in individual cylindrical chambers for 30 min before testing. Thermal nociception was measured using a standard analgesia meter (PL-200; Chengdu Techman Software Co., Ltd., China). Paw withdrawal latency (PWL) in response to radiant heat was recorded to evaluate thermal hyperalgesia, with a 40-s cutoff to avoid tissue injury. Each hind paw was tested five times at 5-min intervals, and the mean PWL of the last three trials was used for analysis (Chen et al., 2024).

### Transcriptomic sequencing and bioinformatics analysis

Total RNA was isolated from the ACC of mice in control and CFA-induced inflammatory pain groups (n = 3 per group) using TRIzol reagent (Thermo Fisher, USA) in accordance with the manufacturer’s instructions. RNA purity was tested using a NanoDrop spectrophotometer, with only samples showing OD260/OD280 ratios of 1.8–2.2 used for further experiments. RNA integrity was determined using an Agilent 2,100 Bioanalyzer, and only samples with RIN ≥ 7.0 were used for library construction. mRNA was enriched via oligo-(dT) magnetic beads and fragmented to ∼200 bp. cDNA libraries were constructed by reverse transcription, end repair, adapter ligation and PCR amplification, then sequenced on an Illumina NovaSeq 6000 to generate 150-bp paired-end reads. Raw reads were filtered using Fastp (v0.23.2) to remove adapters and low-quality sequences. Clean reads were mapped to the mouse reference genome GRCm39 with HISAT2 (v2.2.1). Gene expression was quantified by RSEM (v1.3.3) and normalized to FPKM. Differential expression analysis was performed using the edgeR package (v3.40.2). Genes with FDR < 0.05 and |log2FC| ≥ 1 were considered significantly differentially expressed.

### Molecular docking

The three-dimensional structure of OLFR482 was predicted using AlphaFold Server. The structure of linalyl acetate (LA) was downloaded from PubChem (CID: 8294) and energy-minimized under the MMFF94 force field. Molecular docking was performed using AutoDock Vina 1.2.3. The receptor was prepared by removing water molecules, ions, and small molecules using PyMol 2.5.2. A docking grid box was set to enclose the entire protein. Ligand and receptor were converted to PDBQT format using ADFRsuite 1.0. Docking was performed with an exhaustiveness of 32; other parameters were kept as default. The top-scoring binding pose was selected for subsequent analyses. Visualization was done with PyMol 2.5.2.

### Molecular dynamics simulation

The docked complex was used as the initial structure for all-atom MD simulations using AMBER 24. The AM1-BCC charges for LA were calculated with the antechamber module. The ligand and protein were described by the GAFF2 and ff14SB force fields, respectively. The system was solvated in a truncated octahedral TIP3P water box with a 10 Å buffer, and Na^+^/Cl^−^ ions were added to neutralize the system. Energy minimization was performed using 2,500 steps of steepest descent followed by 2,500 steps of conjugate gradient. The system was then heated from 0 K to 298.15 K over 200 ps under constant volume, followed by 500 ps of NVT equilibration and 500 ps of NPT equilibration. A production run of 100 ns was carried out under NPT conditions (1 atm, 298.15 K) with periodic boundary conditions. Non-bonded interactions were cut off at 10 Å, long-range electrostatics were treated with the Particle Mesh Ewald (PME) method, and bonds involving hydrogen atoms were constrained using the SHAKE algorithm. Temperature was controlled with a Langevin thermostat (collision frequency γ = 2 ps^-1^), and pressure was maintained at 1 atm. The integration time step was 2 fs, and coordinates were saved every 10 ps for analysis.

### RNA fluorescence *in situ* hybridization (FISH)

To preserve RNA integrity and cellular architecture, freshly dissected ACC samples were cleaned in PBS (Servicebio, China) and fixed in ISH fixative (Servicebio) at 4 °C overnight (16–18 h). The fixed tissues were subsequently dehydrated through a sucrose gradient—consisting of 8 h in 15% (w/v) sucrose followed by overnight incubation in 30% (w/v) sucrose until precipitation occurred. Cryosectioning was performed on a Leica CM1950 instrument to yield 10–12 μm sections, which were adhered to microscope slides. Section preparation was finalized by a 30-min ambient drying step, a 10-min secondary fixation in 4% PFA (Servicebio), and triple PBS washes (5 min per wash). Upon reaching ambient temperature, tissue sections underwent an 8-min incubation with 20 μg/mL protease K (Servicebio, China) to facilitate cellular permeabilization. Subsequent washes with nuclease-free water and PBS were conducted to eliminate any residual enzyme activity. To block non-specific target sites, slides were then subjected to a 1-h pre-hybridization step within a humidified chamber at 40 °C, utilizing pre-warmed pre-hybridization buffer (Servicebio, China). Hybridization was initiated by applying 100 μL of hybridization solution (Servicebio, China) containing the *Olfr482*-specific probe and sealing with coverslips. Slides were incubated overnight at 40 °C in a dark humid chamber to allow probe-target annealing. On the following day, sections were washed twice with preheated 2× SSC buffer (40 °C) for 15 min each, followed by a 10-min wash with 0.1× SSC buffer to remove unbound probes. After gentle air-drying, sections were incubated with preheated branch probe hybridization solution (Servicebio, China) at 40 °C for 45 min in a humidified chamber to amplify fluorescence signals, followed by three washes with preheated washing buffer (40 °C) to eliminate non-specifically bound amplifiers. For nuclear counterstaining, sections were incubated with DAPI (1 μg/mL; 4′,6-diamidino-2-phenylindole, Servicebio) for 5 min protected from light. Following PBS rinses, the sections were mounted using an anti-fade mounting medium (Servicebio), and coverslips were sealed with clear nail polish to prevent desiccation. Fluorescence imaging was conducted on a Nikon Eclipse Ni-U upright microscope coupled with a DS-Ri2 camera, utilizing 40× or ×60 oil immersion objectives. All probes were synthesized by Servicebio and validated for specificity via BLAST analysis against the mouse genome.

### Stereotaxic virus injection

Mice were anesthetized with 2% isoflurane and fixed in a stereotaxic frame (RWD Life Technology, China). Ophthalmic ointment was applied to protect the eyes. After scalp shaving and disinfection with iodine and 75% ethanol, a midline incision was made to expose the skull. Bregma and lambda were visualized after removing overlying connective tissues and rinsing the skull with saline. Bilateral craniotomies (∼1 mm diameter) were drilled at the ACC coordinates relative to bregma: AP +1.2 mm, LM ±0.4 mm, DV −2.0 mm. Virus was injected at 30 nL/min via a micropipette (WPI) connected to a microsyringe pump. The pipette was retained for 5 min post-injection to avoid backflow, then slowly retracted. The scalp was sutured with 6–0 silk sutures, and topical antibiotic ointment was applied. Mice were recovered under a heat lamp and monitored closely.

### Whole-cell patch-clamp recording

In experiments involving LA treatment, LA (100 mg/kg) was administered intraperitoneally 30 min prior to slice preparation. Modified protocols were used to prepare 300 μm-thick coronal mouse brain slices containing the ACC while preserving neuronal viability. Slices were immediately transferred to oxygenated artificial cerebrospinal fluid (ACSF) with the following composition: 124 mM NaCl, 25 mM NaHCO_3_, 2.5 mM KCl, 1 mM KH_2_PO_4_, 2 mM CaCl_2_, 2 mM MgSO_4_, and 10 mM glucose. Throughout the entire process, ACSF was continuously aerated with a gas mixture of 95% O_2_ and 5% CO_2_ to maintain a stable pH and oxygen supply. Prior to electrophysiological recording, slices were incubated at 32 °C for 1 h to allow recovery from the slicing procedure. Electrophysiological recordings were conducted under an Olympus microscope equipped with IR-DIC optics, which enabled clear visualization of individual neurons in the ACC. A whole-cell patch-clamp technique was employed using an Axon 200B amplifier (Axon Instruments, USA) to record neuronal activity. Glass micropipettes with a resistance of 3–5 MΩ were pulled using a pipette puller and backfilled with an internal solution (pH 7.2) consisting of 145 mM potassium gluconate, 5 mM NaCl, 1 mM MgCl_2_, 0.2 mM EGTA, 10 mM HEPES, 2 mM MgATP, and 0.1 mM Na_3_GTP. Pyramidal neurons in the ACC were specifically identified based on their typical morphological features (pyramidal soma and distinct dendritic arborization) and characteristic firing patterns during current-clamp recordings. For action potential (AP) recordings, GABAergic synaptic inputs were blocked by adding 100 μM picrotoxin to the bath solution. In contrast, spontaneous excitatory postsynaptic currents (sEPSCs) were recorded in the presence of 1 μM tetrodotoxin (TTX) to inhibit voltage-gated Na^+^ channels and eliminate action potential-dependent synaptic transmission. All electrophysiological signals were digitized at a sampling rate of 10 kHz using pCLAMP 10.7 software (Axon Instruments). To ensure data reliability, continuous monitoring of series resistance was performed during recordings, and any experiment with a series resistance change exceeding 15% was excluded from the final data analysis.

### Statistical analysis

GraphPad Prism served as the platform for all statistical analyses. Each dataset underwent prerequisite assessments for Gaussian distribution and variance equality. Data presentation defaults to mean ± s. e.m., with any exceptions explicitly stated in the text. Statistical significance (P < 0.05) was determined using the two-tailed tests specified in the figure captions, which incorporated necessary *post hoc* controls for multiple testing.

## Data Availability

The data presented in the study are deposited in the NCBI repository, accession number PRJNA1482286. The BioSample accession numbers for six mouse ACC samples are SAMN61119118, SAMN61119119, SAMN61119120, SAMN61119121, SAMN61119122, SAMN61119123.
